# It's easier to get along with the quiet neighbours

**DOI:** 10.15252/msb.20177961

**Published:** 2017-09-26

**Authors:** Laurence D Hurst

**Affiliations:** ^1^ The Milner Centre for Evolution Biology and Biochemistry University of Bath Bath UK

**Keywords:** Chromatin, Epigenetics, Genomics & Functional Genomics, Genome-Scale & Integrative Biology

## Abstract

Why are neighbouring genes co‐expressed at the RNA level? While it is tempting to think that this is to enable coordination of functionally related proteins, analysis of human proteomics data by Rappsilber and colleagues (Kustatscher *et al*, 2017) suggests this is the exception not the rule. Rather it might either be just something that happens or, in some instances, an epiphenomenon of coordination of expression to enable reduced gene expression noise.

Since the earliest explorations in post‐genomics, one truth seems universal—genes in proximity in a genome tend to be co‐expressed (Hurst *et al*, 2004), at least when considering mRNA levels. Why is this? An appealing explanation is that functionally related genes whose protein products are needed at the same time, evolve to be close in the genome enabling coordinated regulation. A recent paper by Kustatscher *et al* (2017) puts a final nail in the coffin for this hypothesis, at least as an explanation for most mRNA‐level co‐expression in humans. They show only for a few rare gene pairs is co‐expression at the mRNA level translated into protein‐level co‐expression. For most genes, RNA‐level co‐expression is dampened at the protein level (Fig 1). This, however, they suggest, does not mean that the close gene coupling and co‐expression is not without utility or consequence.

**Figure 1:**
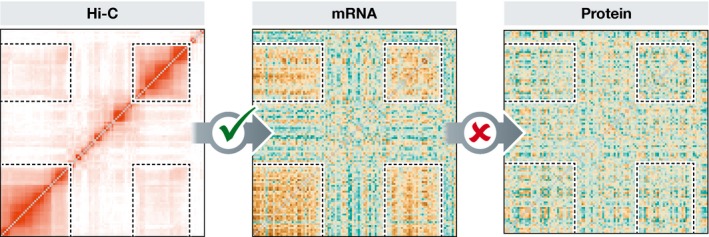
Genes interacting at 3D level are co‐expressed at the mRNA level but not at the protein level The figure illustrates an example of patches of genes that are spatially proximal in the 3D structure of the human genome (Hi‐C data), are co‐regulated at the mRNA level but are not co‐regulated at the protein level (Kustatscher *et al*, 2017).

Genes in a bidirectional orientation and in very close proximity (< 1 kb) account for an unexpectedly high proportion (~10%) of gene pairs in our genome (Trinklein *et al*, 2004). Such genes tend to be co‐expressed at the RNA level and seen together as a pair in the mouse genome (Trinklein *et al*, 2004). It could be supposed that the co‐expression of the two genes in such a pair enables protein‐level coordination. To test this idea, Kustatscher *et al* (2017) examined housekeeping genes expressed in human lymphoblastoid cell lines and, unusually, examined both RNA‐ and protein‐level co‐expression. Looking at 167 such very close bidirectional gene pairs, they found 31 showing strong co‐expression at the RNA level. Of these, only three gene pairs showed sustained co‐expression at the protein level. Generally, those buffered at the protein level do not share common functions, while those retaining co‐expression do (Kustatscher *et al*, 2017). The same RNA‐level co‐expression and protein‐level attenuation are seen for genes in close proximity not in bidirectional orientation. These results suggest that the protein‐level functional co‐operation model for gene order evolution explains some, but not very much, of the observed genome organization, concurring what prior evidence in yeast.

Then why are so many genes so tightly coupled in bidirectional orientation (Trinklein *et al*, 2004) and why are so many neighbouring genes co‐expressed to some degree? An attractive null model to explain neighbour co‐expression proposes that open chromatin affects blocks of genes and enables their expression (Raj *et al*, 2006). Kustatscher *et al* (2017) add to this null model showing that genes that are co‐expressed at the mRNA level tend to reside in the same or interacting genome “compartments”, identified in Hi‐C data as showing long‐range interactions. Importantly, this is true for neighbour genes and genes not in the immediate 1D vicinity but that nonetheless are interacting in 3D space. While this co‐expression does not translate to the protein level, the authors sensibly suggest that there is no reason to suspect any active buffering. Post‐transcriptional effects such as different miRNA regulation, different ribosome occupancy and different protein half‐lives are probably enough to explain why an initial signal of mRNA‐level co‐expression disappears at the protein level. Quantitative modelling of the expected collapse of co‐expression, from RNA into protein level, for random gene pairs would help clarify whether the attenuation is any more than would be expected. One enigma is why genes that interact in 3D are functionally more similar than expected by chance (Thevenin *et al*, 2014) but apparently not co‐expressed at the protein level.

While this RNA co‐expression of co‐compartment genes does not translate to the protein level (Fig 1), Kustatscher *et al* (2017) also report that genes whose protein products are co‐expressed do not correlate with spatial organization of the DNA. For these functionally related proteins, the authors suggest that post‐transcriptional adaptations may well be required to preserve co‐expression (e.g. selection for similar protein half‐life). It is indeed striking that unlinked genes whose proteins are part of the same protein complex have stronger correlations at the protein level than at the RNA level (Kustatscher *et al*, 2017).

So perhaps co‐expression is mostly just a product of chromatin dynamics and, with very few exceptions, there is nothing to write home about. Kustatscher *et al* (2017), however, argue that there is most likely more to it than this. Indeed, if gene expression parameters are modulated at a chromatin level or by local interactions, gene order might well evolve in response to such effects, assuming selection to be strong enough. In this context, a key further property of genomes is the observed clustering of housekeeping/essential genes (Lercher *et al*, 2002; Pal & Hurst, 2003). Co‐expression of essential genes is not so important but since essential genes are, by definition, dose‐sensitive, it is important to ensure that their dose never sinks to low levels by chance. Indeed, as expected, essential genes have low noise in expression and so rarely risk the calamitous fate of accidentally being depleted (Batada & Hurst, 2007).

In this context, it was suggested that genomic organization on both a cluster (Batada & Hurst, 2007) and gene‐pair (Wang *et al*, 2011) level might be part of the solution to the problem of noise control for dose‐sensitive genes. Keep chromatin permanently open and transcription is always possible. Allow genes to maintain each others’ open chromatin status and expression level fluctuations can be smoothed. The effect can be most profound for genes in bidirectional orientation and genomically very close: one gene primes the neighbour (and vice versa) and in so doing stabilizes expression levels (low noise) (Wang *et al*, 2011). Toy models of this mutual interdependence predict that RNA co‐expression is an epiphenomenon of gene pair dynamics of low noise genes (Wang *et al*, 2011).

Preliminary data supported this showing that essential gene clusters are indeed low noise clusters (Batada & Hurst, 2007), that transgenes inserted into such low noise clusters adopt a low noise level (Chen & Zhang, 2016), that genes in bidirectional orientation have especially low noise (Wang *et al*, 2011) and that essential genes are especially likely to be found in bidirectional orientation (Wang *et al*, 2011). Kustatscher *et al* (2017) importantly show that, indeed, for highly gene dense regions gene clustering is associated with reduced noise at both RNA and protein levels, as this model predicts. Gene density is here potentially important as gene–gene non‐independence is to a first approximation dependent on between‐gene distance profile (Wang *et al*, 2011).

Thus, there seem to be three viable models to account for pervasive co‐expression of neighbouring genes at the mRNA level. First, for a very limited number of neighbouring gene pairs co‐expression *per se* is an important part of their biology, these pairs being especially highly co‐expressed, functionally related and, at least in yeasts, preserved as a pair over evolutionary time. Second, many other signals of co‐expression are better considered a happenstance of chromatin dynamics. But, third, some co‐expression appears to viably be explained as an epiphenomenon of selection for low noise of dose‐sensitive genes, by adoption of the dynamics of DNA that enable co‐expression. What is unclear is how common this adoption is and whether, for example, it can explain why gene density varies around a genome. Are housekeeping clusters high‐density clusters to enable this sort of noise control by enabling non‐independence in expression? Or might we instead be looking at a mutation bias? For reasons still unclear, essential gene clusters in yeast have unusually low recombination rates (Pal & Hurst, 2003). Could a relationship between recombination and sequence gain/loss dynamics explain such a trend?
